# Control Work

**Published:** 1896-03

**Authors:** 


### CONTROL WORK.

An outbreak of rabies is reported at Hazleton, Pa., involving the loss of a large number of dogs, some hogs, and a few cattle, all of which were bitten.

*National.* Under date of February 1st the Secretary of Agriculture has issued a circular of instruction concerning the regulations of cattle transportation, specially suggested by the Act of Congress approved March 2, 1895. The lines of quarantine applying to splenic or Texas fever are denoted, commencing in California and ending at the northeast corner of Virginia where it joins Maryland. Special conditions may be allowed by the Department for individual States wishing to adopt other quarantine lines, these changes only to be for special periods, subject at any time to change and cease to exist at the end of period for which the same is granted, from the fifteenth day of February to the fifteenth day of November during each year. No cattle are to be transported from said area below the quarantine line to any portion of the United States above, except by rail for immediate slaughter, and these only under the following conditions:

1. When unloaded above said line to be fed and watered only at certain designated places set apart for this purpose.

2. At the points of destination only in certain pens, no other cattle to be allowed in the latter, and State regulations at such points to be carefully observed. Cars to be thoroughly disinfected before being again used.

3. Cars carrying such cattle shall be labelled Southern cattle, and way-bills so marked, the same rules to apply when reshipped in other cars and way bills so designated as before, and when unloaded must be in pens set apart for these animals.

4. Instructions for disinfection of cars, boats, and pens:

*a.* Remove all litter and manure; disinfect the same by mixing it with lime or saturating with a 5 per cent. solution of carbolic acid. If not disinfected to be stored in a safe place until after November 15th.

*b.* Wash the cars, feeding and watering troughs with water until clean.

*c.* Saturate the walls, floors of cars, fencing, troughs, and chutes of the pens with a solution of chloride of lime, four ounces to the gallon of water. Or disinfect the cars with a jet of steam under a pressure of not less than fifty pounds to the square inch.

Cattle from Mexico may be admitted into the United States to remain below the quarantine line after inspection according to law, but said cattle shall not be permitted to cross said quarantine line otherwise than by rail for immediate slaughter, except by special permit of inspectors of Bureau of Animal Industry under regulations of the Bureau, said permit only to be granted to cattle free from splenic fever or contact thereunto during the three months preceding the issuance of said permit, and which have been grazed in a locality free from infection of such fever.

During the first eight months of 1893, 10,177; the same period of 1892, 15,614; of 1895, 22,755 head of horses were shipped abroad.

The growing value of this trade and the value of the same to our people, will be given further encouragement and support by a thorough inspection and tagging for identification so that there will be no opportunity to check its growth upon any alleged sanitary grounds.

Two experimental grass stations have been established under the Department of Agriculture, to better study the value and methods of cultivation of foreign plants and grasses.

*Massachusetts.* The abolishment of the Cattle Commission in Massachusetts was a topic for discussion at a meeting of some ten of the granges, when the following statement was made by one J. L. Harvey, of Lumenburg, that babies fed on tuberculous milk thrive wonderfully, that nine out of ten people think the Cattle Commission unsatisfactory, and that 59½ per cent. of last year's expenses were office expenses, and that stock is injured by the use of tuberculin. Many of those present opposed the remarks, and favored letting the Commission alone, and that farmers make a great mistake in opposing the Commission. Reports of large number of cattle tested in Vermont and parts of Massachusetts were brought forward, all of which showed the reliability of the tuberculin-test. Where the animals were destroyed, in one herd, where 26 cases out of 36 were killed, and physical examination only revealed 8 out of the 26, which were taken away from the herd some time previously. Subsequently the herd was tested with tuberculin, when the number reached 26, all of which proved tuberculous when destroyed. Eighteen out of 24 of these cases had tuberculosis of the udder. The herd was a very valuable one, and entailed a very heavy loss.

The Massachusetts Board of Agriculture, after considering the future work of the Tuberculosis Commission, unanimously adopted the following proposition as modifications of the present law: "That after June 1, 1896, tuberculin should be used as a diagnostic agent on all animals of all herds in which one or more tuberculous animals have been found; 2d. That owners of said herds shall be required to make such changes in their stables and stable-management as shall be necessary to secure to their cattle the conditions known to be essential to health; 3d. Full compensation for animals found to be tuberculous upon any inspection subsequent to the first shall be allowed only when it appeared that the owner had faithfully carried out the changes which he had been desired to make."

Dr. F. H. Osgood, Chairman of the Cattle Commission, recently addressed the Massachusetts Board of Agriculture, at Dalton, on "Rational Stable Management," taking up two of the essentials necessary for the accomplishment of this, viz., a better water-supply and a sufficient quantity of purer air. Among other statements he said that hard water may cause skin diseases; water containing sulphuric acid causes bone diseases; lime causes various troubles, and that almost all contagious diseases may be communicated through the water-supply. His comparison of the air of stables compared with that of houses, that all the exhalations of the animal's body, together with the discharges from the bowels and kidneys, which must add very much greater impurities to the air, and which in stables adhere to the woodwork and walls, giving rise to what is usually referred to as stable odor. He thoroughly laid bare in his remarks the absurd idea, which is often expressed, that emanations from manure pits are not unhealthy, and made many valuable pungent remarks on stable hygiene.

---

In another part of the JOURNAL will be found a decision in a case of great importance to every practising veterinarian, especially those in large cities.

